# Sex-specific influence on cardiac structural remodeling and therapy in cardiovascular disease

**DOI:** 10.1186/s13293-019-0223-0

**Published:** 2019-02-04

**Authors:** Elise L. Kessler, Mathilde R. Rivaud, Marc A. Vos, Toon A. B. van Veen

**Affiliations:** 1 Department of Medical Physiology, Division of Heart and Lungs, University Medical Center, Utrecht, Utrecht University, Yalelaan 50, 3584CM Utrecht, The Netherlands; 2 Department of Experimental Cardiology, Division of Heart and Lungs, University Medical Center, Utrecht, Utrecht University, P.O.Box 85500, Heidelberglaan 100, Utrecht, 3584CT The Netherlands; 3 Department of Clinical and Experimental Cardiology, Heart Center, Academic Medical Center, Amsterdam, The Netherlands

**Keywords:** Sex, Cardiovascular disease, Hypertrophy, Fibrosis, Inflammation, Apoptosis

## Abstract

**Background:**

Cardiovascular diseases (CVDs) culminating into heart failure (HF) are major causes of death in men and women. Prevalence and manifestation, however, differ between sexes, since men mainly present with coronary artery disease (CAD) and myocardial infarction (MI), and post-menopausal women predominantly present with hypertension. These discrepancies are probably influenced by underlying genetic and molecular differences in structural remodeling pathways involved in hypertrophy, inflammation, fibrosis, and apoptosis. In general, men mainly develop eccentric forms, while women develop concentric forms of hypertrophy. Besides that, women show less inflammation, fibrosis, and apoptosis upon HF. This seems to emerge, at least partially, from the fact that the underlying pathways might be modulated by estrogen, which changes after menopause due to declining of the estrogen levels.

**Conclusion:**

In this review, sex-dependent alterations in adverse cardiac remodeling are discussed for various CVDs. Moreover, potential therapeutic options, like estrogen treatment, are reviewed.

## Background

Although sex-differences in heart failure (HF) and underlying cardiovascular diseases (CVDs) is not an unexplored topic, sex-based therapies are still remarkably scarce. Independent of the injury, CVDs lead to adverse cardiac remodeling, which can eventually culminate into HF. HF is classified based on selected symptoms, as described for example by the Task Force of the European Society of Cardiology, and the left ventricular ejection fraction (LVEF) separating into HF with preserved LVEF (> 50%, HFpEF), HF with moderate LVEF (40–50%, HFmEF), and HF with reduced LVEF (< 40%, HFrEF) [1]. Epidemiology and pathophysiology of CVDs and therefore HF often differ between sexes [2, 3]. Until now, sex-based discrepancies in the essential molecular pathways of CVDs such as coronary artery disease (CAD), myocardial infarction (MI), hypertension, valvular heart disease, chronic obstructive pulmonary disease (COPD), and different forms of cardiomyopathy (namely, arrhythmogenic (ACM), hypertrophic (HCM), and dilated (DCM)) are incompletely understood, aggravating the development of proper treatment for both sexes.

Many of these sex-differences are caused by the presence or absence of sex hormones, but besides sex hormones of course also other factors can influence adverse cardiac remodeling in a sex-specific manner, such as sex chromosomes and epigenetics. It has been shown that several genes related to, e.g., hypertension and adverse cardiac remodeling processes, such as macrophage activation, apoptosis, and lipid metabolisms are located on the Y chromosome [4]. Moreover, lack of the X chromosome can lead to various diseases as is further elaborated on in the discussion, and incomplete X chromosome inactivation can lead to disease progression as reviewed in, e.g., Regitz-Zagrosek and Kararigas [5]. However, the role of the chromosomes and epigenetics, as reviewed by, e.g., Hartman et al. [6], is beyond the scope of this review.

Also metabolism, pregnancy, life-style, and environmental factors play an important contributing role on sex-differences in the most common CVDs. These influences have been reviewed extensively before [5, 7, 8]. Therefore, our review specifically aims at summarizing sex differences in the molecular remodeling mechanisms, which are particularly involved in hypertrophy, inflammation, apoptosis, and fibrosis.

### Sex differences in patients with CVD

When generalized, prevalence of CVDs and overall mortality are independent of sex [3]. However, epidemiology and pathophysiology of CVDs often differ as has been extensively reviewed in literature and is briefly summarize below and in Table 1.

**Table 1:** Sex differences in cardiovascular structural remodeling

	Myocardial infarction	Hypertension/aortic stenosis/PO	Pulmonary hypertension/COPD	HCM/DCM	ACM
Prevalence	M > F in all age groups [9, 18]	F < M pre-menopausal, F > M post-menopausal [14, 31]	F > M Pulmonary hypertension [86] F > M COPD [17]	M > F in all age groups [18]	M > F in all age groups [18]
Mortality	M = F [3, 9]	depending on age [3]	M > F [1]	M > F in all age groups [18]	M > F in all age groups [18]
Hypertrophy	M > F [21, 44]	M: eccentric F: concentric [1, 14, 22, 25]	M > F [38, 39]	M > F [69, 87]	M > F [18]
Inflammation	M > F [48]	M > F [34]	M > F [88]	M > F [69, 89]	Sex unmentioned [90]
Fibrosis	M > F and M larger infarct size [44]	M > F [14, 34, 63]	M > F [67]	M > F [69]	No significant differences in fibro-fatty replacement [71]
Apoptosis	M > F and M larger infarct size [62]	M > F [64]	M > F [67]	M > F [69]	M > F in iPS cells [72]

Abbreviations: *F* = female; *M* = male; *ACM* = arrhythmogenic cardiomyopathy; *COPD* = chronic obstructive pulmonary disease; *DCM* = dilated cardiomyopathy; *HCM* = hypertrophic cardiomyopathy; *PO* = pressure overload

CAD and MI are more prevalent in men than in women in all age groups (43% in men versus 27% in women in the CONFIRM Long-Term Registry) and women acquire those diseases about 10 years later in life, though overall mortality has shown to be equal specifically for CAD and MI (about 50% in Writing Group 2016) [3, 9, 10]. In the CONFIRM study, women who presented with CAD were older (62.4 years versus 58.9 years in men) and suffered more from hypertension (60% versus 52% in men) [9]. After MI, women often develop HFpEF (37% versus 23% in men), whereas men mainly present with HFrEF [11]. Prevalence of hypertension keeps increasing and is the most treated risk factor for CVD and HF, leading to mainly HFpEF in women and HFrEF in men [12]. Pre-menopausal women have a lower incidence of hypertension than age-matched men (e.g., 7.3% versus 15.8% men, between 30 and 44 years of age) [13]. This difference vanishes during menopause until the age of 65, when more women present with hypertension than men. This fact is mainly thought to be caused by the influence of estrogens in females, but might also partly be explained by a genetic component as is suggested through the identification of a hypertension associated region on the Y chromosome in animals [4, 14, 15]. Reports on valvular heart diseases are contradictory, indicating that more women suffer from mitral valve disease (male:female ratio, 3:1), and depending on the different studies, either more men or women present with aortic valve disease (male:female ratio, 3:1 or 0.76:1) [3, 14].

Pulmonary hypertension causes right ventricular (RV) cardiac remodeling, with pulmonary stenosis and COPD being the two main contributors. In contrast to systemic hypertension, pulmonary hypertension is more prevalent in younger women (< 65 years) than in men (male:female ratio, 1:1.8 in the UK), but pregnancy in younger women highly contributes to these numbers. However, at older ages, the prevalence equalizes [16]. In both sexes, the disease often culminates into HFpEF, and men present with a higher mortality, despite the lower prevalence [1]. In the non-smoking group, about 80% of COPD patients are female [17].

Prevalence of the different forms of cardiomyopathies, such as DCM, HCM, and ACM is higher, average age of diagnosis is lower, and outcome is worse in men than in women (male:female ratio of DCM 1.5:1, of HCM 3:2, and of ACM 3:1) [18]. Interestingly, inherited forms of these cardiomyopathies, e.g., due to mutations in troponin T (for DCM), Plakophilin-2 (for ACM) and in sarcomeric proteins (for HCM), also show a skewed incidence, suggesting that on top of the genetic predisposition, additional factors are decisive for the phenotypical expression of the disease [18].

**Fig. 1:**
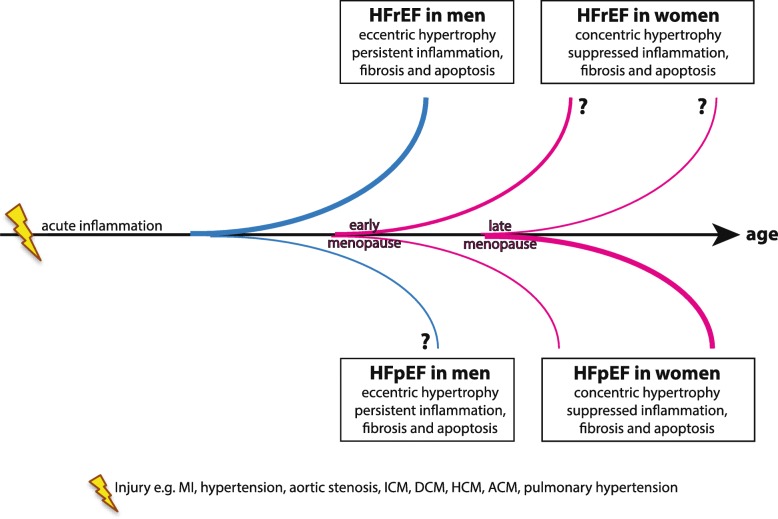
Generalized development of sex differences in structural cardiac remodeling and possible heart failure (HF) outcomes. Men (blue lines) and women (red lines) often show different structural remodeling after an initial injury (e.g., hypertension and MI.) leading to acute inflammation and different disease outcomes. In general, women tend to develop HF later than men. The older, the more patients suffer from cardiovascular diseases; however, in women this seems to be clearly related to the onset of their menopause. Thickness of lines mirrors the general number of patients suffering from the HF type at that age

Regardless of the cardiovascular injury, women tend to develop HFpEF, while most men develop HFrEF (e.g., 73.4% males developed HFrEF and 48% developed HFpEF in the PREVEND study [19]) as depicted in Fig. 1. Whether women developing HFrEF or men developing HFpEF show the same characteristics of cardiac remodeling as their counterparts is currently still under investigation (indicated in with a question mark).

### Sex differences in molecular pathways of adverse cardiac remodeling

CVDs trigger adverse cardiac remodeling, which is manifested by hypertrophy, inflammation, fibrosis, and apoptosis, eventually leading to HF. Many of these processes are often sex-specific (see Table 1) and influenced by sex-hormone levels of estrogen, testosterone, or progesterone [20]. Obviously, men and women are on different hormonal schedules, and although the hormonal shifts during a woman’s life are more frequent and dramatic than in a men’s life, it is thought that the effects of sex-hormones, in particular estrogen, during the pre-menopausal period are able to delay the appearance of CVDs in women [15]. This is supported by the observation that women with irregular hormone cycles and an early menopause have an elevated risk of HF [20].

In the following sections, we will discuss the mentioned mechanisms and processes triggering adverse cardiac remodeling in the light of sex-specific differences.

#### Hypertrophy

The development and manifestation of cardiac hypertrophy differs between the sexes. Generally, in women, LV remodeling due to MI or aortic stenosis/hypertension is oriented towards concentric hypertrophy leading to diastolic dysfunction (Fig. 1 pink lines). In men, eccentric hypertrophy with chamber dilatation is the most common, eventually leading to systolic dysfunction (Fig. 1 blue lines) [1, 21, 22]. Next to that, the amount of viable cardiomyocytes decreases in the healthy aging male heart more extensively than in the female heart, making it at baseline already more susceptible to stress and hypertrophy [23]. As mentioned, this effect in females is thought to be, at least in part, due to estrogens and estrogen receptor (ER) signaling, which is especially mediated by the estrogen receptor beta (ER-β) [24]. Studies in animal models support this. Mice lacking the ER-β develop hypertrophy faster, and females with aortic stenosis show a more extensive upregulation of ER-β, while ER-α is upregulated to the same extent in both sexes [25, 26]. Estrogen-treated ovariectomized (OVX) female mice show a slower development of LV hypertrophy compared to untreated OVX animals [27]. In patients, estrogen is associated with a better RV systolic function, and androgens, such as testosterone, with an increased RV mass and volume, as is seen during hypertrophy [28]. Moreover, estrogen is known to modulate natriuretic peptides, e.g., pro-brain-type natriuretic peptide (BNP) and activates angiogenesis, which supports the hypertrophic heart in its elevated oxygen demand [29]. Estrogen has also been shown to temper hypertrophic remodeling through CaMKII inhibition, which adds to the preserved EF in pre-menopausal women [30].

Pre-menopausal women have a lower incidence of hypertension than age-matched men as mentioned earlier. This is in line with observations that estrogen can modulate β-adrenergic signaling and may cause vasodilation by increasing endothelial nitric oxide synthase (eNOS) [31]. LV pressure overload leads in general to activation of the neuro-hormonal axis and to an increase of the sympathetic drive. In young men, peripheral resistance is proportional to sympathetic drive, though not in young women. Post-menopausal peripheral resistance increases with sympathetic drive [32]. This is probably due to the decrease of estrogen, since baroreflex sensitivity is modulated by estrogens [33]. Because of that, post-menopausal women have a higher prevalence of hypertension and pulmonary hypertension. In addition, men with aortic stenosis show a more robust upregulation of genes related to the renin–angiotensin–aldosterone system (RAAS), whereas women are able to repress the gene encoding for angiotensinogen [34]. Transverse aortic constriction (TAC), mimicking aortic stenosis in mice and rats, shows in the majority of studies that female animals develop HF later than males, accompanied with a less dilated phenotype [35].

Although estrogen is known for its vasodilator properties, pulmonary hypertension is more prevalent in younger women, and the role of estrogens and testosterone in pulmonary hypertension remains controversial. After hypoxia-induced pulmonary artery hypertension in mice with overexpressed serotonin transporters, OVX leads to a reduced RV hypertrophy, whereas estrogen therapy (ET) re-established the hypertension phenotype [36, 37]. In contrast, in rats with pulmonary hypertension, experimentally induced by, e.g., monocrotaline or bleomycin, estrogen has been reported to be beneficial against RV hypertrophy, inflammation, and fibrosis, and females show a less severe phenotype than males [38]. Besides that, these different outcomes might be related to differences in the type of rodent and the method used to induce hypertension; in numerous studies, female animals show less pulmonary hypertension than male animals as reviewed in Mair et al. [39]. However, the same investigators suggest that men are probably more protected from pulmonary hypertension due to the fact that the vasodilatory effects of testosterone are more important in the pulmonary vasculature than that of estrogens [39].

HCM presents by definition with concentric and DCM with eccentric hypertrophy. Both disorders are more prevalent in men as discussed before. Also in mice, females seem to develop less DCM and HCM than males, and later in life upon end-stage HF induced by chronic LV pressure overload [18]. ACM leads to predominantly RV dilatation in humans and mice (e.g., upon Plakophilin-2 knockout) [40], but also biventricular phenotypes are known [41]. In ACM, dilatation may be caused by increased stretch of the cardiac walls and be more visible in the right ventricle, since this wall is significantly thinner [42]. In men, this effect is often more pronounced, which might be due to a higher intrinsic sympathetic drive and activity in young men and male children [43].

Although studies in animals highlight potential mechanisms of cardiovascular remodeling mediated by sex-hormones, these models often make use of one sex-hormone at a time and are therefore not optimal representations of the fine balance between different sex-hormones under physiological conditions. Extrapolations to the human condition should therefore be considered with care.

#### Inflammation

Inflammatory pathways contribute to the progression of adverse remodeling and are activated upon cardiac injury. As mentioned before, the Y chromosome contains several genes involved in, e.g., macrophage activation [4]. Higher numbers of neutrophils and macrophages in males lead to an increased extent of inflammation, and together with a reportedly higher activity of matrix metalloproteinases (MMPs), this can cause premature breakdown of collagen and therefore cardiac rupture and slowed healing after injury or during HF [44].

**Fig. 2:**
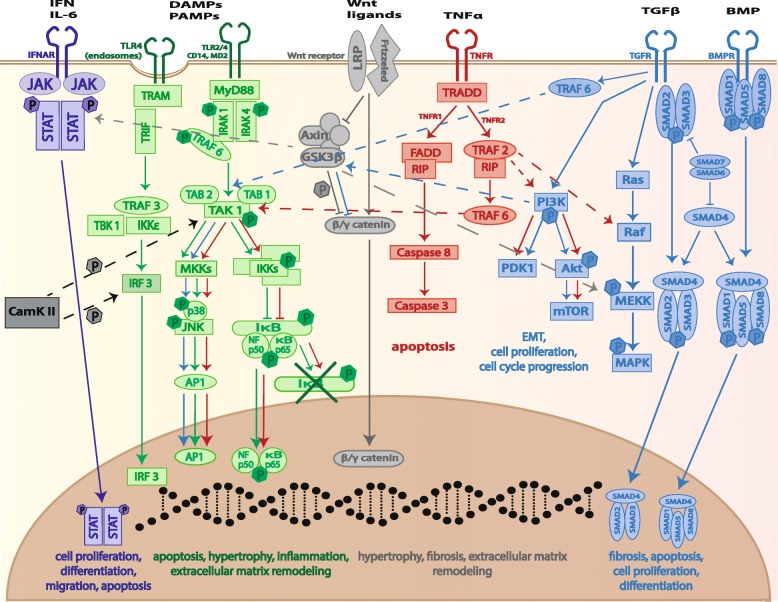
Molecular signaling of inflammatory pathways involved in structural remodeling. Inflammatory pathways activated upon cardiac injury include the IFN-γ/JAK-STAT pathway (purple), the TLR pathway (green; either in an endosome (left pathway) or on the cell membrane (right pathway)), the TNF-α/MEKK pathway (red), and the TGF-β/BMP/SMAD pathway (blue), triggering secretion of cytokines and chemokines and leading to, e.g., apoptosis and cell proliferation. Crosstalk between the pathways is possible (dashed arrow) and collaboration with the Wnt pathway (light gray) and CaMKII (dark gray) promoting cardiac inflammation. Sex differences in these pathways are discussed in the text, such as the inhibitory effects of estrogen on CaMKII, BNP, and NFκB

The most common inflammatory pathways triggering secretion of cytokines and chemokines and subsequently the attraction of inflammatory cells like dendritic cells and macrophages are depicted in Fig. 2 and discussed in the following paragraphs.

##### The JAK-STAT pathway

Binding of a ligand to the interferon (IFN) receptor leads to activation of the Janus kinase-signal transducer and activator of transcription (JAK-STAT) pathway, causing translocation of a STAT dimer to the nucleus (Fig. 2, purple pathway). A study showed that in women undergoing LV adverse remodeling due to pressure overload, genes of the JAK-STAT pathway are significantly downregulated [34]. Mechanical strain upon hemodynamic stress (such as in TAC animal models) induces production of the cytokine interleukin-6 (IL-6), activating the JAK-STAT pathway, and also the toll-like receptor (TLR) pathway (Fig. 2, green pathway) [45]. Female mice lacking the ER-β have significantly increased activation of inflammatory signals, such as IL-6, and presence of ER-β seems to prevent cardiac inflammation and fibrosis in mice upon TAC [25, 46].

##### The toll-like receptor pathway

The inflammatory TLR pathway activation is associated with adverse cardiac remodeling, arrhythmias, and HF [47]. Binding of a ligand to a TLR (either in an endosome (Fig. 2, left green pathway) or on the cell membrane (Fig. 2, green pathway)) leads to either translocation of IRF3 or AP1 to the nucleus, or degradation of the inhibitor of NFκB (IκB) and consequently transport of NFκB to the nucleus. In women, the TLR pathways are to a lesser extent activated than in men, and male patients show increased expression of various inflammatory proteins upon LV remodeling (e.g., chemokine receptor-1, IL-33, and TLR7) [34]. In animals with experimentally induced hypertension and MI, TLR expression is increased in males, while knockout of TLRs (e.g., TLR4 and TLR2) improves cardiac function more in males than in females after MI [48–51]. Interestingly, ER-α can act as a transcription factor, which can inhibit expression of NFκB [52].

Crosstalk between different remodeling mechanisms is noticed since hypertrophy can interact with inflammatory signaling, where CaMKII (which is prominently activated during adverse remodeling) can directly phosphorylate components of the TLR pathway, thereby promoting cardiac inflammation (Fig. 2, black arrow) [53]. As mentioned above, estrogens can interact with CaMKII and members of the TLR pathway, therefore playing an important part in this interaction. In the future, therapeutic interference with these pathways, as exampled by TLR blockers, should therefore take the sex of the patient and his/her hormone levels into account.

##### The Wnt pathway

During inflammation, the Wnt pathway (Fig. 2, gray pathway) is activated and known to regulate T cell development, dendritic cell, and leukocyte maturation [54]. Activation of the Wnt pathway leads to degradation of the GSK3-β complex resulting in distortion of catenins from the membrane and translocation to the nucleus. Men with aortic stenosis show a larger upregulation of the Wnt pathway than women [34]. Crosstalk between the Wnt/β-catenin and the TLR pathway has been reported, where depletion of β-catenin leads to increased levels of TLR4 [54]. In ACM patients, mutation of γ-catenin (also known as Plakoglobin) or β-catenin causes weakening of intercellular junctions, probably leading to detachment of cardiac cells. This causes cell death and necrosis, which has been associated with inflammation and eventually fibrosis formation, although sex differences in this regard have not been investigated [55]. Not only interactions between GSK3 and ER-α, but also androgen receptors have been reviewed in detail [56], and this should be taken into account when investigating for example GSK3-β blockers. Furthermore, Wnt signaling is involved in a plethora of (developmental) processes, making it a difficult target for therapeutic interventions.

##### The TNF pathway

Binding to the tumor necrosis factor (TNF) receptor activates TRADD (Fig. 2, red pathway), which in turn either activates FADD and the apoptotic caspase pathway or TRAF2, which leads to cross activation of the TLR (green pathway) and TGF-β and bone morphogenic protein (BMP) pathways (Fig. 2, blue pathway). Also upon MI, the apoptotic pathway is activated by caspase-11 that in turn can activate caspase-1. The latter is able to induce interleukin (e.g., IL-1α, IL-6) and TNF-α production, and their secretion causes activation of more inflammatory cells and pathways, such as the TLR and MEKK pathway [57]. Estrogen has been shown to inhibit the synthesis of TNF-α, thereby hampering the activation of inflammatory cells and fibroblasts [58]. This suggests that in women, the TNF pathway is less active compared to men and therefore the development/progression of CVDs might be slowed. Repressing the TNF pathway therapeutically might therefore have a greater benefit in men and post-menopausal women than pre-menopausal women.

##### *The TGF-*β and BMP pathway

Invading inflammatory cells, such as macrophages, express the TGF-β receptor, which is able to mediate the inflammatory response [59]. Activation of the TGF-β or BMP receptor leads to SMAD complex formation, activation of Ras and the mitogen-activated protein kinase pathway, or activation of PI3K (Fig. 2, blue pathways). This eventually results in epithelial to mesenchymal transition or activation of the mTOR pathway causing changes in cell cycle and proliferation. Women show lower expression of TGF-β and a lower inflammatory response [34] probably since ER-β binding by estrogen on fibroblasts has been shown to block TGF-β and AngII stimulation [46]. In addition, TGF-β is highly pro-fibrotic and macrophages play a role in transition of fibroblasts to myofibroblasts; the latter being more pro-fibrotic in their activity [59]. This illustrates the tight connection between inflammatory and fibrotic responses in cardiac remodeling, logically leading to less of both in women, or at least a delay in response when compared to men.

Concluding, all cardiac inflammatory pathways can be modulated by estrogen and therefore, sex differences should be taken into account when conducting research on and/or testing therapeutic interventions on CVDs.

#### Fibrosis and apoptosis

During all forms of CVDs and HF, and in both sexes, cardiac fibroblasts secrete excessive amounts of collagen into the extracellular matrix. While collagen genes are upregulated in all kinds of human CVDs and rodent disease models, Metalloproteinases (MMPs) are often downregulated or unchanged, and Tissue Inhibitors of MMPs (TIMPs) are either upregulated or also unchanged, as thoroughly reviewed in Fan et al [60]. Besides, although different CVDs result in different patterns of fibrosis, its formation and deposition appears later in the process of remodeling in women as compared to men [34].

Apoptosis is the programmed cell death following, e.g., cardiac injury, and in general, female failing hearts show lower rates of apoptosis and necrosis than males. This could, on the one hand, be due to the presence of apoptosis-related genes on the Y chromosome, or on the other hand, although not completely understood, due to the role of estrogen and ERs [4].

After MI in mouse models, mostly compact fibrosis is found due to massive apoptosis and necrosis in the infarcted area, which is more extensive and results in more collagen content in male mice compared to females [61]. This is further confirmed in humans, where apoptosis after MI is more prevalent in men compared to women [62]. After pressure overload, female rodents present with lower levels of apoptotic proteins, such as caspase 3 and 9, and ER-β signaling attenuates apoptosis and seems to prevent cardiac fibrosis upon chronic pressure overload and in HF [25, 63, 64]. In OVX rats, estrogen therapy is able to reduce collagen content and MMP/TIMP balance upon LV pressure overload and apoptotic protein levels [64, 65]. In addition, as already explained, females show lower levels of TGF-β and angiotensinogen, both involved in synthesis and deposition of fibrosis [34]. Male patients undergoing aortic valve surgery, on the other hand, display increased levels of collagens I and III, and MMP expression as compared to female patients [66]. In male HFpEF patients, also expression of TIMP-2 appeared to be increased, as compared to females [34]. Pulmonary hypertension leads to RV fibrosis and apoptosis in both sexes [67]. Reports on the role of sex-hormones in pulmonary hypertension are contradictory: since prevalence is higher in females, one would suggest a protective role for, e.g., testosterone. However, castrated rats with pulmonary hypertension showed the opposite since decreased RV fibrosis was observed while administration of testosterone lead to an increase in collagen content [68].

Women with DCM have less fibrosis and apoptosis, and show a more reversible type of HF in clinics [69]. In DCM patients with lamin A/C mutations, nuclear accumulation of androgens such as testosterone has been found, and castrated mice carrying the same mutations showed improved cardiac function and less cell death and fibrosis [70]. Structural sex-based differences in the clinical manifestation of ACM, such as the disease-characteristic fibro-fatty replacement and apoptosis, have not been reported [71], probably since detection is predominantly based on MRI which allows only the detection of larger areas of fibrosis. However, in a recent study, testosterone worsened and estradiol improved the degree of cardiac apoptosis and lipogenesis in an ACM model of induced pluripotent stem cells [72]. Moreover, lipid metabolism seems to be associated with loci on the sex chromosomes, possibly leading to sex-specific differences in fibro-fatty replacement [4].

Apoptosis and fibrosis in the myocardium cause structural abnormalities and inhomogeneity of the syncytium, which is an important factor that affects efficiency of contractility and predisposes to arrhythmogenesis [73]. Therefore, delayed deposition of fibrosis and apoptosis in female hearts as compared to males might postpone the appearance of such cardiac dysfunctions and should be targeted in future research in a sex-specific manner.

### Sex differences in therapeutic interventions

Pharmacological interventions for CVDs and HF include prescription of statins, neuro-hormonal blockage by, e.g., angiotensin-converting enzyme (ACE) inhibitors, angiotensin receptor blockers and β-blockers, and instrumentation via, e.g., an implantable cardiac defibrillator (ICD) in order to prevent sudden death caused by arrhythmic events. In general, except for diuretics, most drugs are more often prescribed in men than in women, even after adjusting for age and mortality. Often absorption, distribution, metabolism, and clearance differ between sexes and sex-specific differences have been reviewed in, for instance Tamargo et al. [74], and are shortly summarized in Table 2. Unfortunately, current guidelines are not considering these differences and therapeutic interferences like neuro-hormonal blockage are to a lesser extent beneficial in patients with HFpEF and therefore, in general, less effective in women than in men [3]. Moreover, women have a lower distribution volume for medications, such as β-blockers, related to differences in body dimensions and fat distribution, and often show a lower clearance rate [75]. Prescription of drugs varies between sexes, where statins, for example are more likely prescribed to men than women (64.8% in men versus 57.6% in women). Furthermore, of all ICD implantations, only 17–26% are performed in women [76–78]. The latter most likely can be explained by the fact that guidelines for ICD therapy (LVEF < 35%) are based on the results of clinical trials, where men are overrepresented.

**Table 2:** Sex differences in therapeutic options for CVDs

Therapeutic option	Effect
Statins	M = F [78], but F higher risk of adverse drug reaction
ACE inhibitors	M = F [74]
Angiotensin receptor blockers and β-blockers	M = F, but F slower clearance, lower volume of distribution, and less sensitive [74]
ICD	M = F [76, 77]
Calcium channel blockers	F faster clearance of, e.g., verapamil and nifedipine, but faster reduction of blood pressure [74]

Abbreviations: *F* = female; *M* = male; *ACE* = angiotensin-converting enzyme; *ICD* = implantable cardiac defibrillator

Pharmacological treatment for pulmonary hypertension mainly includes calcium channel blockers, whose mode of action can be increased in women due to higher bioavailability [16, 74].

Therapy for ACM aims at preventing arrhythmias and reducing the rate of SCD with, e.g., exercise restrictions and ICD therapy [41]. Since ICD therapy in ACM is often used as primary prophylaxis, sex-biased outcomes are not that prominent.

Since many differences exist between the response and therapies, and therefore their efficiency between women and men, it is surprising that there are no sex-specific guidelines to treat CVDs and HF. Most of the therapeutic options are driven by the manifestation of CVDs in men, while they are also the primary cause of death in women [1]. Future sex-specific therapeutic options should therefore focus on the underlying differences in cardiac remodeling, but one should also keep in mind that often the primary injury seems to be already different between sexes. This is, e.g., the case in CAD, where women show more often microvascular and men macrovascular dysfunction [10]. Furthermore, biomarker reference intervals for these diseases have not historically been established for men and women separately, which may partially contribute to the gaps in underlying diagnosis.

This together illustrates that sometimes a different treatment-regimen will be needed for men and women.

Estrogen therapy (ET) has been investigated as a potential treatment for CVDs in post-menopausal women as extensively reviewed in Miller and Harman [79]. In rodents with chronic pressure overload, ET seems to increase EF and reduce fibrosis formation [80]. However, controversial results in clinical trials using ET show either improvement of contractile performance upon, e.g., hypertension or an actual increase in the risk of stroke, MI, and pulmonary embolism [81]. The effect might be dependent on the type of estrogen administered (e.g., differently dosed 17-β estradiol (1 or 2 mg), estradiol valerate, conjugated equine estrogen, or combined therapies), the way of administration (orally or non-orally), and the timing/frequency. Besides that, positive effects of ET are often only seen in clinical trials, if estrogen is administered within 1 year after menopause suggesting that it is not possible to reverse some permanent alterations during disease progression (e.g., fibrosis or accumulation of atherosclerotic plaques) [82].

Furthermore, in “extreme patients,” such as individuals with congenital Turner syndrome, the effect of lack of estrogen can be highlighted. Women with Turner syndrome lack one X chromosome partially or fully and often suffer from premature ovarian failure leading to estrogen deficiency or even depletion [83]. These women often present with hypertension are at risk for various cardiac diseases and have a 3-fold increased mortality. Hormone replacement therapy and ET is a standard therapy that is already initiated early in life. ET in the affected women does not only normalize, e.g., uterine size, but was also shown to improve blood pressure and, at a high dose, increased the cardiac intima media thickness as intensively reviewed in Cintron et al. [83]

In line with that, women with congenital Kallmann syndrome are characterized by hypogonadotropic hypogonadism and anosmia. Moreover, they often suffer from congenital heart diseases. ET is also used in these cases already early in life to induce secondary sex characteristics, but less is known about its potential role in cardioprotection [84].

## Conclusions and future perspectives

Although sex-specific discrepancies are seen in almost all CVDs, appropriate diagnosis and consequent therapies are not well established. This is not only partly due to severe underrepresentation of women in clinical trials (young women were even banned from clinical trials by the FDA from 1977 to 1993) [85], but also caused by the lack of knowledge on molecular disparities between sexes with regard to cardiac remodeling pathways for hypertrophy, inflammation, fibrosis, and apoptosis. In this review, we summarized the knowledge on sex-dependent differences in these pathways and the involvement of sex-hormones, especially estrogen. Overall, women show less fibrosis, hypertrophy, and inflammation upon CVDs than men, often preserving their EF. This leads to an onset of a most often different phenotype of HF later in life, but on the other hand prevents women from receiving the appropriate timely treatment, since the absence of a proper HF diagnosis means that no treatment will be provided. This increases the risk of sudden cardiac death in females. Therefore, also women with HFpEF should be treated or at least be monitored to reduce mortality in this group.

Moreover, women seem to seek less cardiac health care, treatment (such as an ICD) is often less well perceived and induces more psychological discomfort (depression and stress) than in men, and men are more often heart donors and receivers [14, 35]. Partially, this could rely on the fact that different cardiac symptoms are experienced by women and men, which could hamper the recognition in women that they actually are experiencing, e.g., an MI. These facts demonstrate that sex-specific differences in the underlying mechanisms of structural remodeling indeed lead to differences in CVDs and health care and should be more thoroughly investigated to advance and specify treatment.

## Abbreviations

ACE: Angiotensin-converting enzyme
ACM: Arrhythmogenic cardiomyopathy
BNP: Pro-brain-type natriuretic peptide
CAD: Coronary artery disease
CaMK II: Calmodulin kinase 2
COPD: Chronic obstructive pulmonary disease
CVD: Cardiovascular disease
DCM: Dilated cardiomyopathy
ECM: Extra cellular matrix
eNOS: Endothelial nitric oxide synthase
ER: Estrogen receptor
ET: Estrogen therapy
ET-1: Endothelin-1
HCM: Hypertrophic cardiomyopathy
HF: Heart failure
HFmEF: Heart failure with moderate LVEF
HFpEF: Heart failure with preserved LVEF
HFrEF: Heart failure with reduced LVEF
ICD: Implantable cardiac defibrillator
IFN-γ: Interferon-γ
IL: Interleukin
JAK-STAT: Janus kinase/signal transducer and activator of transcription
LV: Left ventricle
LVEF: Left ventricular ejection fraction
MEKK: Mitogen-activated protein kinase kinase
MI: Myocardial infarction
MMP: Matrix metalloproteinase
MRI: Magnetic resonance imaging
NF-κB: Nuclear factor kappa-light-chain-enhancer of activated B cells
OVX: Ovariectomized
PLN: Phospholamban
RAAS: Renin–angiotensin–aldosterone system
RV: Right ventricle
SCD: Sudden cardiac death
TAC: Transverse aortic constriction
TGF-β: Transforming growth factor-β
TIMP: Tissue inhibitor of MMP
TLR: Toll-like receptor
